# A national survey of foundation dentists’ and educational supervisors’ experiences: part 1 – motivations, achievements, and challenges

**DOI:** 10.1038/s41405-026-00433-0

**Published:** 2026-05-12

**Authors:** Elizabeth Gonzalez Malaga, Sana Movahedi, Shalin Mehra, Vanessa Elaine Muirhead

**Affiliations:** 1 NHS England Workforce, Training and Education (WT&E), London, UK; 2 NHS England Workforce, Training and Education, Thames Valley and Wessex, UK; 3 Centre for Dental Public Health and Primary Care, Institute of Dentistry, Faculty of Medicine and Dentistry, Queen Mary University of London, London, UK

**Keywords:** Dental foundation training, Health care

## Abstract

**Introduction:**

Completing Dental Foundation Training (DFT), guided by Educational Supervisors (ESs), is the route that almost every new UK dental graduate takes to work as independent NHS primary care performers. However, little is known about their achievements, motivations, and challenges. Few studies have included ESs.

**Aims:**

This study assessed reasons why Foundation Dentists (FDs) pursued DFT and the motivations of ESs. It examined their perceived achievements and challenges during DFT.

**Methods:**

An anonymous online survey was sent to FDs and ESs in England, Northern Ireland and Wales in February - April 2024 about their motivations, achievements, and challenges.

**Results:**

469 FDs (52% response rate) and 640 (71% response rate) ESs participated. Most (91%) FDs wanted to build their clinical confidence, and ESs (98%) wanted to explore career opportunities and share knowledge (94%). While 96% of FDs felt that DFT had enhanced their clinical skills, 62% faced challenges providing complex care. ESs (37%) had difficulty managing trainees’ performances. Six percent and 4% of FDs had experienced bullying and harassment from patients, and ESs or the dental team respectively.

**Conclusions:**

DFT provided opportunities for new graduates to develop clinical skills and confidence. Actions should address challenges in DFT related to providing complex clinical care and bullying and harassment.

## Introduction

Dental foundation training (DFT), previously known as dental vocational training (VT), was introduced in 1977 as a voluntary initiative to support the transition from dental school to independent clinical practice [1]. Since 1993, almost all new UK dental graduates have completed a minimum of one year of DFT in England, Wales or Northern Ireland, or VT in Scotland, before they work as independent performers in NHS primary care in general dental practice or salaried services [2]. Dentists from non-UK dental schools and UK dental graduate schools with more than 12 months of experience can join the NHS Performer list through alternative routes [3].

DFT enables foundation dentists (FDs) to develop as competent, caring, reflective, safe and effective primary care practitioners [4]. The DFT curriculum is a competency-based framework covering four key domains: clinical, communication, professionalism, management, and leadership. The ‘Dental Gold Guide’ sets out the key objectives covering a range of competencies including improving clinical skills in routine and complex care, understanding primary care, identifying strengths and weaknesses, promoting patients’ oral health, and enabling personal development and training [5].

Foundation dentists (FDs) are also supported by educational supervisors (ESs) who are experienced dentists, with more than four years of dental practice experience. ESs directly supervise, educate, mentor, and assess FDs aiming to create supportive and positive training environments [6]. As mentors, ESs inspire their trainees to drive positive change in patient care and the profession by ‘modelling good professional behaviour and attitudes at all times’ [7].

Despite the long history of DFT, little is known about the experiences of FDs or ESs and about their perceptions about the benefits of DFT [8]. Aside from anecdotal evidence, there is limited information on what motivates FDs to complete DFT besides beyond obtaining an NHS Performer number, the challenges they face during this critical training period, and the motivations and challenges experienced by ESs in their roles. Addressing these key questions is an essential step needed to identify strategies to enhance the experience of FDs and identify any needed support and training for ESs. Moreover, understanding the challenges and early clinical practice experiences of FDs is particularly important given the NHS Long Term Workforce Plan and proposal to introduce a ‘tie in for newly qualified dentists’ [9]. This could require newly qualified dentists to spend a minimum period of time delivering NHS dental care post-qualification.

### Aims

This publication is part 1 of a series initiated by the UK Committee of Postgraduate Dental Deans and Directors (COPDEND) to provide a comprehensive national evaluation of DFT, including perspectives from FDs and ESs. The task was assigned to the Dental Foundation Training Advisory Group (DFTAG), an advisory group under COPDEND, that oversees all FDs and ESs in England, Northern Ireland, and Wales. The objectives of this first paper were to: (i) identify the reasons why FDs chose to complete DFT and ESs chose to become supervisors (ii) assess FDs’ perceptions about achieving the Dental Gold Guide competencies (iii) and explore the difficulties encountered by FDs and ESs during DFT. Part 2 of the series will cover differential experiences encountered by FDs in relation to their protected characteristics.

## Materials and methods

This was a cross-sectional study involving FDs and ESs across England, Northern Ireland and Wales who joined the September 2023 DFT cohort. Vocational Trainees from Scotland were excluded because of the differences in training structures and NHS dental contracts. As no validated questionnaires exist, two anonymous online Microsoft Forms surveys were specifically developed using the aims and objectives set for Foundation Dental training in the Dental Gold Guide, which includes 11 domains [5]. The FD survey contained 17 questions, including a multiple-choice question about their reasons for choosing to complete DFT. Five-item Likert agreement questions captured their perceptions on their clinical skills, understanding primary care, identifying personal strengths and weaknesses, promoting patients’ oral health and providing quality dental care, carrying out audits and peer reviews to support continuing professional development (CPD), decision-making and referrals and applying ethical standards. Yes/no questions captured the difficulties related to completing Work Based Assessments (WBAs), providing routine and complex care, communicating with patients, ESs and dental teams, and experiencing bullying and harassment from patients, ESs, and dental teams adapted from the NHS England National Education and Training Survey (NETS) [10]. Free-text questions enabled FDs to provide additional information.

The ES survey included 19 questions. Contextual information was collected about the time years spent as ESs and the type of dental practice (NHS, mixed, practice). ESs were also asked about the reasons for choosing to become ESs and the difficulties related to managing FDs’ clinical performance, attending study days and other training and communicating with the communicating with NHS Workforce, Training and Education (NHS WT&E) team.

Both surveys included demographic questions related to the following protected characteristics: age, sex, ethnic group, disability, sexual orientation, religion, marital status, and pregnancy status. No information about region was collected to maintain confidentiality for the anonymous survey.

The surveys were pre-tested by COPDEND and DFTAG. All FDs and ES were sent a link to the surveys through their work email addresses circulated by the Deaneries. The survey was open from 1 February to 30 April 2024. Two reminder invitations were sent to FDs and ESs.

The survey responses were imported as an EXCEL file and the data analysed using the Statistical Software for Social Science (SPSS) version 29 to produce descriptive statistics and summaries. Even though the survey was anonymous, all figures are presented as percentages to prevent disclosure. Content analysis was used to analyse the free-text responses.

As this project was an evaluation of education and training under the auspices of COPDEND with oversight and approval, ethical approval was deemed as not required based on the Health Research Authority (HRA) definition of research [11]. This was a voluntary survey of dental professionals and consent was implied by completing the survey.

### Ethics declaration

This paper adhered to the principles set out in the Declaration of Helsinki, 2024. Institutional ethical approval was not required for this survey carried out by the UK Committee of Postgraduate Dental Deans and Directors (COPDEND).

## Results

Nine hundred and five FDs were sent the survey and 469 FDs responded (52% response rate). Six hundred and forty ESs participated in the survey (71% response rate).

### Characteristics of FDs and ES survey participants

Table 1 summarises the demographic characteristics of the FDs. As expected, most FDs (59%) were under 25 years of age. Sixty-five percent of FDs were female, consistent with GDC registrant data. Forty two percent were Asian, 31% were White, 5% were from an other ethnic group, 4% were Black, and 2% were mixed. Sixteen percent chose not to disclose their ethnicity. Only three percent of FDs chose to disclose that they had a disability. Forty-one percent of ESs were aged between 35 and 44 years old and 57% of ESs were male (Table 2). Most ESs were White (43%) or Asian (39%).

**Table 1:** Characteristics of dental foundation trainees who participated in the survey (*N* = 469).

Protected characteristics	Percent
Age group	
Under 25	59.3
25–29	31.3
30–35	4.1
Above 36	0.6
Missing (Unknown)	4.7
Sex	
Female	64.8
Male	31.1
Missing (Unknown)	4.1
Ethnic group	
Asian	42.4
Black	3.8
Mixed	2.1
Other ethnic group	4.7
Prefer not to say	6.4
White	30.9
Missing (Unknown)	9.6
Disability	
No	82.7
Yes	3.0
Prefer not to say	3.8
Missing (Unknown)	10.4

**Table 2:** Characteristics of Educational Supervisors who participated in the survey (*N* = 640).

Protected characteristics	Percent
Age group	
25–34 years	13.3
35–44 years	41.3
45–54 years	29.5
55–64 years	12.2
65 and over	1.6
Missing (Unknown)	2.2
Sex	
Female	41.1
Male	57.0
Missing (Unknown)	1.9
Ethnic group	
Asian	39.1
Black	2.8
Mixed	1.7
Other	1.4
Prefer not to say	5.0
White	42.5
Missing (unknown)	7.5
Disability	
No	86.4
Yes	1.7
Prefer not to say	3.9
Missing (Unknown)	8.0

### Reason for FDs choosing to complete DFT and ESs motivations

Improving their confidence and clinical experience (91%) was the most common reason for FDs for choosing to complete DFT (Table 3). Other common reasons included being accepted onto the NHS performers list without conditions (84%) and obtaining a certificate of Satisfactory completion of DFT (79%) (Table 3).

**Table 3:** Reasons why trainees chose to complete dental foundation training (DFT).

FDs’ reasons to complete DFT (Multiple choices allowed)	Number (%)
Acceptance onto the NHS Performers List without conditions	392 (83.6)
Gain a certificate of satisfactory DFT completion	371 (79.1)
Gain experience of the NHS as an internationally qualified dentist	170 (36.2)
Improve confidence and clinical experience	425 (90.6)
Meet the requirements to apply for a Dental Core Training post	152 (32.4)
Gain research experience as an Academic Dental Foundation trainee	35 (7.5)
Other reasons^a^	4 (0.8)

^a^Other reasons included live in UK, requirement for Royal Navy, mentoring, and confidence for private work.

Most (98%) ES were motivated to take on their role to explore career opportunities, to diversify their clinical work schedule (93%), share their knowledge with dental graduates (94%), to network with colleagues (86%) and to develop teaching and training competencies (81%).

### FDs’ perceptions about achieving the Dental Gold Guide competencies

Most FDs felt that DFT had helped them to understand how to promote their patients’ oral health and provide high-quality dental care (98%) and enhance their clinical skills (96%). FDs felt that their DFT had helped them to identify their personal strengths and weaknesses (96%) and to apply ethical practice guidelines (96%) (Table 4). The lowest rated achievement was their ability to carry out audits and peer reviews to support their CPD (72%) (Table 4).

**Table 4:** Trainees’ perceptions about achieving the dental gold guide foundation dentist objectives.

Trainees’ perceptions about achieving the Dental Blue Guide DFT objectives by protected characteristics	Strongly agree/agree Number (%)
I have been able to practice and improve my clinical skills	452 (96.4)
I have been introduced to all aspects of dental practice in primary care	400 (85.3)
I can now identify my personal strengths and weaknesses	449 (95.7)
I know how to promote the oral health of my patients and provide quality dental care	460 (98.1)
I have carried out audits and peer reviews to support my Continuing Professional Development	339 (72.3)
I can confidently make decision about my patients including making decision about referring to other services	403 (85.9)
I can apply practice guidelines to maintain confidentiality and ethical standards	449 (95.7)

### Challenges and difficulties experienced by FDs and ESs

Completing complex dental care to patients (62%) was the most common challenge reported by FDs (Table 5). This concurred with ESs where 37% had experienced difficulties managing the clinical performance of their trainees.

**Table 5:** Challenges reported by trainees’ during their dental foundation training (DFT).

Challenges reported by trainees’ during their dental foundation training	Number (%)
Difficulties completing work-based assessments	114 (24.6)
Difficulties completing routine dental care to patients	395 (14.3)
Difficulties completing complex dental care to patents	289 (61.9)
Difficulties communicating with patients	103 (22.1)
Difficulties communicating with Educational Supervisor	68 (14.7)
Difficulties working with the dental team	39 (8.4)
Difficulties with bullying and harassment from patients	29 (6.3)
Difficulties with bullying from Educational Supervisor or dental team	16 (3.5)
Difficulties with discrimination from patients	26 (5.6)
Difficulties with discrimination from the dental team	11 (2.4)
Difficulties with handling patient complaints	32 (6.9)

A quarter (25%) of FDs had difficulty completing WBAs and 22% had challenges related to communicating with patients. Other difficulties included challenges delivering routine dental care (14%), communicating with ES (15%) and working with the dental team (8%) (Table 5). Seven percent of FDs also reported difficulties with handling patient complaints. Six percent of FDs had experienced bullying and harassment from patients while four percent had experienced from ES supervisors or the dental team. FDs had also experienced discrimination from patients (6%) and 2% from the dental team.

## Discussion

While previous surveys such as the NHS England National Education and Training Survey (NETS) [10] have evaluated FDs training experiences, this study used concomitant surveys of FDs and ESs to explore their motivations, achievements, and challenges. Both surveys found novel findings and insights to support recommendations and strategies to support FDs and ESs.

A key finding was that most FDs chose to complete DFT to improve their confidence and clinical experience to consolidate and further develop their skills. This desire coexisted alongside their awareness about their most common challenge: completing complex dental patient care. This challenge was shared by ESs who felt that managing the clinical performance of FDs was also a key challenge. This finding concurs with a recent qualitative study of ESs in South West England [12]. Matabdin et al. found that participating ESs experienced the stress and burden of supervising FDs who lacked confidence and clinical experience [12]. Although ESs recognised from the full text responses that their role extended beyond supervision to include teaching, mentoring, and coaching, their expectations are that DFT is not designed to compensate for the perceived gaps in clinical experience that their FDs had on completing dental school. An alternative and critique of the perception that FDs lack clinical skills and confidence would argue that this is a mirage, which reflects a mismatch between the expectations of FDs and ESs and their contrasting definition of competence. This better ‘in my day’ idiom and comparison conflicts with notions of competence as defined by Chuenjiwongsa et al. who suggests that ‘being competent does not mean that the dental graduates will not need further practice and development’ [13].

The GDC Scope of Practice [14] and the Safe Practitioner Framework [15] for dentists do not differentiate between routine and complex treatments. Instead, these documents outline that dentists are trained and competent to perform non-specialist dental procedures upon graduation. The findings from these surveys concur with the frequent debate and previous research about ‘preparedness for practice’ and insufficient clinical exposure at the undergraduate level [16]. This presents additional challenges to FDs and ESs to be addressed during DFT. However, the purpose of DFT is defined by legislation under the National Health Service (Performers List) Regulation 2004 [17], as ‘enhancing clinical and administrative competence’ rather than addressing any gaps in undergraduate clinical training and exposure to patients. The multifactorial challenges faced by dental schools are well-recognised, including the high costs associated with clinical training (e.g. maintenance of equipment and facilities) and the lack of clinical academic teaching staff [18]. Any explicit change in the purpose and expectations of DFT to bridge any gaps in undergraduate dental training will require legislative change. However, closer alignment and collaboration between dental schools and DFT schemes is one action that could be taken to address this challenge in the absence of legislative change. For example, the London NHS England Workforce, Training and Education region is working closely with regional dental schools to implement outreach programmes for students to gain more experience in primary care as part of their undergraduate training and sharing educators across both settings.

The newly introduced DFT Early Years Curriculum, currently being piloted in certain regions of England, addresses some questions about the responsibilities of ESs as clinical teachers or supervisors. It incorporates Entrustable Professional Activities (EPAs) to assist educators in determining the appropriate level of supervision required by trainees. Based on the type and complexity of treatment, trainees can gradually progress from direct supervision to reactive, distant, and eventually unsupervised levels. Additionally, the curriculum recommends involving other members of the dental team in the assessment process of WBAs. This approach introduces multiple perspectives, helps minimise bias, and alleviates some of the workload for ESs [19].

At the time of the survey, 28% of FDs reported not having conducted audits or peer reviews. Given the essential role of audits in improving patient care, to ensure all trainees gain experience in this area, completing audits, such as radiograph and patient records audits, is a mandatory component of FDs portfolios. However, since the survey was conducted midway through the training period, some trainees may not have yet had the opportunity to complete this requirement.

Another key finding was the bullying, harassment and discrimination experienced by FDs. Despite ongoing efforts by the NHS to address these issues, they unfortunately remain. The findings align with the NETS survey, where eight percent of dental postgraduate trainees in England reported experiencing bullying or harassment by staff within the learning environment [10]. Similarly, a systematic review of resident doctors showed that bullying also varied by age, sex and ethnicity [20]. As the newest and often youngest members of the dental team, FDs may find it challenging to confront and report incidents. To address these concerns, deaneries, training agencies, and providers have made it a priority to highlight the support available for all trainees through the Professional Support Units. This information is often disseminated during inductions, in trainee resource packs, websites and newsletters. In England, FDs also have access to Freedom to Speak Up Guardians, Occupational Health Services and Wellbeing teams through their Lead Employer Provider, independent of their training practice. This marks a significant improvement from previous arrangements, where access was limited to services within the training practice itself, raising concerns about impartiality. Furthermore, drawing on practices from GP training quality assurance, the implementation of mandatory training for training practices and their teams, along with robust quality assurance processes, are essential measures to help safeguard trainee wellbeing.

Part 2 of the series will provide a more detailed analysis of trainees’ experiences based on their protected characteristics.

## Conclusions

This study underscored the essential role of DFT (supervised by ESs) has consolidating clinical skills, competencies, and clinical confidence in FDs. It highlights the challenges faced by FDs carrying out complex dental care and by ESs who experienced difficulties managing their clinical performance during this transitional year. Actions to address clinical competencies through collaborative working between undergraduate training and DFT should be considered. Raising awareness and adopting strategies to tackle bullying and harassment will reinforce the commitment to supporting FDs well-being and DFT experience.

## Data Availability

Data supporting this study are available from the corresponding author upon reasonable request.
